# Long-Term Efficacy of First Line Antiretroviral Therapy in Indian HIV-1 Infected Patients: A Longitudinal Cohort Study

**DOI:** 10.1371/journal.pone.0055421

**Published:** 2013-01-30

**Authors:** Ujjwal Neogi, Elsa Heylen, Anita Shet, Sara Chandy, Ranjani Shamsunder, Anders Sönnerborg, Maria L. Ekstrand

**Affiliations:** 1 Unit of Infectious Diseases, Department of Medicine Huddinge, Karolinska Institutet, Stockholm, Sweden; 2 Division of Clinical Virology, Department of Microbiology, St. John’s Medical College, Bangalore, Karnataka, India; 3 University of California San Francisco, Department of Medicine, San Francisco, California, United States of America; 4 Department of Paediatrics, St. John’s Medical College Hospital, Bangalore, Karnataka, India; 5 Department of Medicine, St. John’s Medical College Hospital, Bangalore, Karnataka, India; Istituto Superiore di Sanità, Italy

## Abstract

**Background:**

Short term efficacy of combination antiretroviral therapy (cART) in resource-constrained settings is comparable to that found in western studies. However, long term data are limited. India has the third largest HIV infected population in the world but the long-term outcome of first line therapy according to the national guidelines has not been evaluated yet. Therefore, we conducted a long-term longitudinal analysis of the efficacy of the national first-line therapy in India from an observational cohort of Indian patients in two different clinical settings.

**Methodology/Principal Findings:**

A total 323 patients who had been on ART for a median of 23 months and achieved virological suppression <100 copies/ml by their study baseline visit, were included and followed for two years. Blood samples were collected every six months for viral load and CD4 count. Drug resistance genotyping was performed when the viral load was >2000 copies/mL. Adherence and treatment interruptions (>48 h) were assessed via self-report. In the studied patients, the median duration of viral suppression was 44 months; 15.8% of patients showed viral rebound, and 2.8% viral failure. Viral rebound or failure was significantly negatively related to perfect adherence (100% adherence and no treatment interruption >48 hrs). Virological re-suppression in the subsequent visit was observed in three patients without any change in therapy despite the presence of key mutations.

**Conclusion/Significance:**

Our study reports for the first time, a good long-term response to the first line therapy for a median of nearly four years although a less than perfect adherence increases the risk for treatment failure and subsequent drug resistance development. The empirical findings in this study also indicate the overall success of the Indian ART program in two different settings which likely are representative of other clinics that operate under the national guidelines.

## Introduction

The attainment and maintenance of sustained viral suppression is the ultimate goal of antiretroviral therapy (ART). The optimal response to therapy is the substantial decrease in viral load (VL) to below the technical specificities of the VL assays which results in the avoidance of emergence of drug resistance mutations [1]. The treatment guidelines in resource rich settings recommend performing VL monitoring every 3–6 months [2]. However, in resource constrained settings like sub-Saharan Africa and India, treatment efficacy is typically monitored through immunological markers such as measurement of CD4+ T cell count, [3] whereas VL monitoring is usually restricted to research settings. Despite this caveat, the short term outcomes of cART in resource-constrained settings are comparable to those in resource-rich settings [4], [5]. The data on long term outcome, however, are limited as most of the studies have been conducted within the first two years after initiation of ART [5].

The National AIDS Control Organisation (NACO) sponsored by the government of India, initiated the National AIDS Control Program (NACP) to provide free ART through established ART-centres in April 2004 [6]. Currently, nearly 384,000 HIV-1 infected individuals are receiving free ART with one non-nucleoside reverse transcriptase inhibitor (NNRTI), either nevirapine (NVP) or efavirenz (EFV), in combination with two nucleoside reverse transcriptase inhibitors (NRTI); zidovudine (AZT) or stavudine (d4T), and lamivudine (3TC) [7].

India has the third largest HIV-1 epidemic in the world with a burden of nearly 2.5 million people, where HIV-1 subtype C is the predominating subtype [8]. A previous study with a small number of patients (n = 40) using generic ART identified a rapid suppression of HIV-1 viral load [9]. However data on long term efficacy of the national first line regimen is lacking.

Therefore, to understand the clinical impact of the Indian national ART guideline, the primary purpose of our study was to investigate long term virological outcome among Indian HIV-1 infected patients on first-line therapy, following initial viral suppression. In addition, we evaluated the factors associated with virological rebound, its consequences, and emergence of drug resistance mutations.

## Materials and Methods

### Study sample

The sample described in this paper was derived from a large observational clinical cohort of 533 patients from two tertiary care hospitals in the southern part of India, which adhere to the national therapeutic guidelines [10], [11]. The criteria used for inclusion in these analyses were: (i) Adult HIV-1 infected patients initiated on first-line ART for a minimum period of 6 months, and (ii) a viral load <100 copies/ml at the time of entry into the study. First line ART included two NRTI and one NNRTI drug. A total 323 patients met these inclusion criteria. The patients were followed up for every 3 months and evaluated for self-reported adherence, adherence barriers and other health behaviours, and lab samples were collected every 6 months for a total follow-up period of 2 years.

### CD4+ T-cell count, viral load and drug resistance genotyping

Blood samples were collected in EDTA tubes. CD4 count was measured using a single platform flow cytometric assay (PCA system; Guava Technologies Inc., Hayward, CA, USA). Viral load was measured by an in-house real time polymerase chain reaction with TAQMAN assay at Molecular Diagnostics and Genetics, Reliance Life Sciences, Mumbai, India. Given the lower detection limit of the VL assay (<100 copies/mL), intermittent viral rebound was defined as a single episode of detectable viral load >100 copies/mL. Virological failure was defined as two successive viral load values >400 copies/mL. Patients with VL >2000 copies/mL were genotyped for drug resistance using in-house techniques [12], [13]. The drug resistance genotyping was successfully performed on 16 patients samples during their first viremia and a further six in the next visit who had viral load >2000 copies/ml. The mutations were interpreted using International AIDS Society updated drug resistance mutations in 2011 (IAS_2011) [14]. The resistance data was not used for treatment decisions.

### HIV-1 subtyping

HIV-1 subtyping was determined using the maximum likelihood (ML) phylogenetic tree using the reference sequences downloaded from Los Alamos Database (www.hiv.lanl.gov).

### Adherence measurements

Self-reported adherence was measured using a Visual Analogue Scale (VAS) which has been found to predict virological failure better than other self-report measures in this setting [15]. In the present study, two types of adherence measurements were combined into one dichotomous “perfect adherence” variable: Percent of prescribed pills taken, assessed with the Visual Analogue Scale (VAS) and Treatment interruptions, defined as having missed medications for more than 48 hours in the past three months. To be classified as “perfectly adherent”, patients had to report 1) taking 100% of prescribed doses in the past three months (past one month at baseline) at each study visit in which they were present and 2) zero treatment interruptions during the 2-year study period.

### Statistical analysis

Differences between groups on continuous variables were examined by independent sample *t*-test or Mann–Whitney *U*-test, while categorical variables were analyzed via frequencies and cross-tabulations, with χ2 tests to assess the significance of the associations. All analyses were conducted in PASW Statistics version 18.

### Ethical Considerations

The study was approved by the Committee for Human Research at University of California, San Francisco, USA and the Institutional Ethical Review Board of St John’s Medical College and Hospital, Bangalore, India. Written informed consent was obtained from all the study participants before enrolling into the study.

## Results

### Patient demographics and clinical characteristics

The demographic and clinical characteristics of the 323 patients at baseline and during the two year follow-up period are presented in Table 1. Almost three quarters of participants were male, and mean (SD) age at baseline was 38 (8.5) years. Most participants (87.9%) were on a NVP-based regimen. The mean (SD) duration of treatment at baseline was 23 (11) months. Median CD4+T cell count at baseline was 370 cells/mm^3^ (IQR: 243–525). During the study period 35.3% (114/323) patients maintained a good immunological profile (CD4 >350 cells/mm^3^) at all waves in which they were present. The median duration of viral suppression was 44 months (IQR 36–54) and 15.8% (51/323) of patients showed intermittent viral rebound without failure during the study period. Viral failure was observed in only 2.8% (9/323) of patients. Among the patients, 75.2% (243/323) was able to maintain ≥95% adherence throughout the study and 47.4% (153/323) were able to maintain perfect adherence (100% adherence by VAS and no treatment interruptions) during the same time (Table 1). Patients with a single episode of viral rebound had a significantly lower mean viral load compared to the failure group during their first viremia (3.2 vs 4.0 Log copies/mL; p = 0.047) (Table 2). Mean CD4-T cell counts during first viral rebound were also low in the viral failure group compared to the viral rebound group (284 cells/mm^3^ vs. 404 cells/mm^3^), but the difference was not statistically significant (p = 0.147) due to lack of statistical power, given the small number of patients (n = 9) with viral failure.

**Table 1:** Patients’ demographic, clinical and laboratory parameters at baseline and whole study period of 2 years.

	Variable	Parameter
**Baseline**	Total number of patients, N (%)	323 (100%)
	Gender, N (%)	
	Male	235 (72.8%)
	Female	87 (26.9%)
	Transgender	1 (0.3%)
	Age, Mean (SD), years	38 (8.5)
	Duration of treatment, Mean (SD), months	23 (11)
	CD4 cell count, Median (IQR), cells/mm^3^	370 (243–525)
	Drug regimen at baseline, N (%)	
	d4T+3TC+NVP	123 (38.1%)
	AZT+3TC+NVP	160 (49.5%)
	d4T+3TC+EFV	21 (6.5%)
	AZT+3TC+EFV	17 (5.3%)
	FTC+TDF+EFV	1 (0.3%)
	DDI+3TC+NVP	1 (0.3%)
Follow-up*	Intermittent viral rebound, N (%)	51 (15.8%)
	Virological failure, N (%)	9 (2.8%)
	Treatment Interruptions, N (%)	29 (9.0%)
	≥ 95% Adherence, N (%)	243 (75.2%)
	Perfect Adherence, N (%)	153 (47.4%)
	Duration of viral suppression Median (IQR), years	44 (36–54)

**Abbreviations**: d4T, Stavudine; 3TC, lamivudine; AZT, zidovudine; FTC, emtricitabine; TDF, tenofovir; NVP, Nevirapine; EFV, Efavirenz; SD, Standard deviation; IQR, interquartile range; N, Number; PVL, plasma viral load; VAS, Visual analogue scale.

* Over the study period of 2 years,

**Table 2:** Clinical and demographic parameters during first viral rebound for viral failure and intermittent viral rebound groups.

Parameter	Viral Failure (n = 9)	Viral Rebound (n = 51)	*p* value^a^
**Age in years at baseline: mean (SD)**	35.4 (6.7)	38.5 (10.1)	0.390
**Male: n (%)**	8 (88.9)	35 (68.6)	0.423
**CD4-T cell count; cells/mm^3^: mean (SD)**	284 (241)	404 (224)	0.147
**Viral load (log transformed): mean (SD)**	4.0 (1.2)	3.2 (1.0)	0.047
**100% adherence past month: n (%)**	5 (55.6)	40 (80.0)	0.195

a p-value based on t-test (d.f. = 58) for age and CD4 count and viral load, Fisher’s exact test (d.f. = 1) for male gender and adherence.

### HIV-1 subtyping

**Figure 1:**
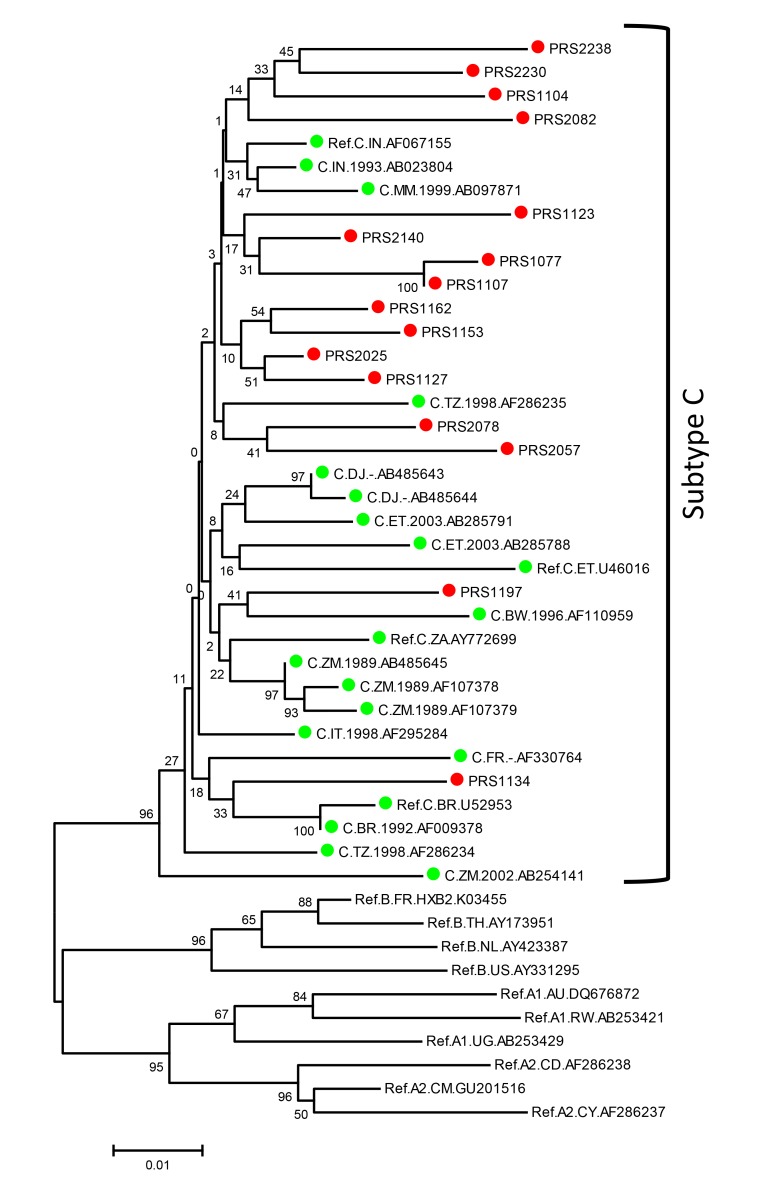
Phylogenetic analysis for characterisation of HIV-1 subtypes: The phylogenetic tree was constructed using general time reversible (GTR) model with gamma distribution and invariant sites (GTR+G+I) as observed best fitted model for the dataset with cohort sequences and reference sequences downloaded from Los Alamos Database (www.hiv.lanl.gov). Subtype C reference sequences from different countries are marked with green filled circle. Cohort sequences are shown with red filled circle.

HIV-1 subtyping using the Maximum likelihood (ML) phylogenetic tree with the best fitting general time reversible substitution model with inverse gamma distribution (GTR+G+I) with 500 bootstrapped data sets identified all the study sequences as HIV-1 subtype C (Figure 1), which is predominant in India [8].

### Correlates of viral rebound/failure

Comparisons between participants with and without viral rebound/failure (Table 3) showed that about half of those without rebound/failure reported maintaining perfect adherence (100% adherence without any treatment interruption) during the study period, compared to less than a third of those who did experience viral rebound/failure (p = 0.007). None of the other variables were significantly associated with viral rebound/failure.

**Table 3:** Viral rebound/failure and its associations with patients’ demographic, clinical, and behavioural parameters.

Variables	Viral rebound/failure (n = 60)	No viral rebound/failure (n = 263)	p value^a^
**Age in years: mean (SD)**	38.0 (9.7)	37.6 (8.3)	0.703
**Male: n (%)**	43 (71.7)	192 (73.0)	0.834
**Mean CD4 –T cell count in cells/mm^3^ over study period: mean (SD)**	410 (217)	420 (221)	0.758
**Regimen switch: n (%)**	29 (48.3)	116 (44.1)	0.553
**Perfect Adherence: n (%)**	19 (31.7)	134 (51.0)	0.007

a p-value based on t-test (d.f. = 321) for age and mean CD4 count, chi-square test (d.f. = 1) for male gender, regimen switch and perfect adherence.

### Drug resistant genotyping

Drug resistance mutations were identified in 9 of the 16 patients with VL > 2000 at their first instance of viremia during the study period (M184V: 8; K70R: 1; K65R: 1; G190A: 4; K103N: 3; Y181C: 3; V108I: 3; H221Y: 2) (Table 4). Among these 16 patients, seven had successfully controlled the viremia in their subsequent 6-month follow up study visit without any change of therapy. Of the seven controlled viremics, three had multiple major NRTI/NNRTI mutations [M184V+G190A; M184V+K103N; M184V+Y181C) during their viral rebound, but later supressed as they maintained >95% adherence both before and after the viral rebound. Further, all three patients maintained good immunological conditions (mean CD4+ T cell count 618 cells/mm^3^) in their remaining study visits for 12 months, 18 months and 6 months, respectively.

**Table 4:** Characteristics of patients (n = 16) with VL > 2000 at first virological rebound/failure.

Patient ID	CD4 count (cells/mm^3^)	VL at rebound (copies/mL)	VL at follow-up (copies/mL)*	Months on ART at viral rebound	Therapy	NRTI Resistance	NNRTI Resistance
1077	33	70500	38850	34	AZT+3TC+NVP	M184V	G190A
1104	711	1144100	<100	53	AZT+3TC+NVP	None	None
1107	895	3750	<100	40	AZT/d4T+3TC+NVP	M184V	G190A
1123	398	2300	1000	36	d4T+3TC+NVP	None	None
1127	74	1699300	NA	38	d4T+3TC+NVP	None	None
1134	214	4900	2950	36	d4T+3TC+NVP	K70R, M184V	V108I, Y181C, H221Y
1153	160	342300	<100	48	d4T/AZT+3TC+NVP	None	None
1162	176	4726550	<100	22	d4T+3TC+NVP	None	None
1197	753	5550	<100	35	AZT+3TC+NVP	M184V	K103N
2025	427	5350	NA	40	d4T+3TC+NVP	None	None
2057	19	3050	NA	47	AZT/d4T/DDI+3TC+NVP	K65R, M184V	K103N, V108I
2078	147	14350	15300	41	d4T+3TC+NVP/EFV	M184V	K103N
2082	21	764100	435100	30	d4T+3TC+NVP/EFV	None	A98G, K101E, V108I, Y181C, G190A, H221Y
2140	560	43000	<100	13	d4T+3TC+NVP	None	None
2230	324	30700	<100	30	AZT+3TC+EFV/NVP	M184V	Y181C
2238	98	333300	62388	29	AZT+3TC+EFV/NVP	M184V	K101E, V106M, G190A

Abbreviations: 3TC, lamivudine; AZT, zidovudine; d4T, Stavudine; DDI, didanosine; EFV, Efavirenz; HAART, highly active antiretroviral therapy; NNRTI, non- nucleoside reverse-transcriptase inhibitors; NRTI, nucleoside reverse-transcriptase inhibitors; NVP, Nevirapine; VL, viral load. *VL follow-up after 6 months.

Among the six patients with virological failure who had high viremia during their first viral rebound, four had accumulated more NRTI drug resistance mutations (DRMs) in their follow up visit (data not shown).

## Discussion

Our results indicate a good overall long-term response to first line therapy reflecting an overall success of the standardized Indian national program for individuals with HIV infection. By the end of the two-year study period, 81.4% of the patients remained virologically suppressed for a total median duration of nearly four years (based on time on ART at baseline + two-year study period) and this efficacy is likely to continue be longer as most of the patients reached the study endpoint with good immuno-virological conditions. As the study was conducted in two different settings, one public tertiary care hospital and one non-profit private tertiary care hospital, which follow the same therapeutic guidelines, recommended by the NACO, it is likely that the results are generalizable to other Indian clinics that follow the same protocols.

Previous studies on short term efficacy of antiretroviral therapy from resource limited settings, within two years after initiation, have obtained comparable results to studies conducted in resource rich settings in Europe and America [4], [5]. A recent systemic review showed that in sub-Saharan Africa, 67% of the patients achieved virological success after 2 years of therapy [5]. However, long term follow up data after 2 years of therapy, are limited. A study from Botswana, estimated virological failure in 22.1% and 30.1% of HIV-1 subtype C infected patients at 3 and 5 years respectively [16]. A study from South Africa reported that 61% of the patients achieved virological suppression at 3 years [17]. A study from Burkina Faso reported that 81.8% of the patients remained virologically supressed at 3 years [18]. Our study showed that 81.4% of the patients who initially achieved viral load <100 copies in our setting, remained virologically suppressed for a median of almost four years, 15.8% had an intermittent viral rebound, and only 2.8% of patients experienced a true virological failure. This study is the first to document the favourable treatment response of a median of nearly four years duration with the national first line treatment regimen in India.

Non-adherence to therapy has been shown to be associated with intermittent viremia [19], [20] in therapy experienced patients. A significant relationship between adherence (including treatment interruptions) and viremia was observed in our study, which is consistent with previous findings in this region [10]. However our study contradicts a recent study from British Columbia, Canada, which showed the probability of viral rebound was lower in patients who had longer duration of viral suppression and was independent of adherence level [21]. The discrepancies in the two settings might be due to the drug regimen used. Thus, our study showed that the risk of viral rebound in perfectly adherent patients is lower than in imperfectly adherent patients. Previous work in our setting has shown that individual-level adherence barriers (away from home, busy with other things, and simply forgetting) and medication side effects are the major obstacles for adherence [11], [15], [22].

Previous studies have shown that sub-optimal adherence, treatment interruptions and improper dosing can lead to drug resistance [10], [23], [24]. A recent meta-analysis on studies from resource limited settings showed that early identification of virological failure limits the emergence of drug resistance [25]. In our study, three patients with virological rebound showed major mutations, including M184V, K103N and Y181C. However, at the next visit, their viral load was suppressed to below the detectable limit. It is important to note that these three patients had good immunological status during that time and good adherence throughout the study periods. Studies from Sweden, Belgium and South Africa showed that despite the presence of NRTI mutations such as M184V, viral re-suppression occurred in a fraction of patients with virological rebound [26]–[29]. Studies on subtype C virus from South Africa showed that despite the presence of NNTRI mutations such as K103N, V106M and G190A, a fraction of patients were successfully re-suppressed on the same regimen [28], [29]. It was also proposed that supporting the adherence with special targeted counselling without any treatment change among such patients could be of cost benefit for the public health system in resource constrained settings. Our finding corroborates this idea. The patients in our settings are under good adherence counselling as part of a standardised national program and maintained an overall good adherence profile throughout the study period. Though 19% of the patients presented viral rebound, only 2.8% of the patients finally failed virologically. This combination of findings by us and others provides support for the conceptual premise that viral load measurement can act as an indicator for adherence. Early identification of viremia and targeted special adherence counselling to these patients with viral rebound may limit the need to switch to the second line of therapy.

In contrast, among the six virologically failing patients who had higher viremia during their first viral rebound, four accumulated new NRTI mutations by their subsequent follow-up visit, which is concordant with a previous finding from South Africa which observed increase in NRTI DRMs in 88% of subtype C infected patients between two visits (6 to 12 months apart) [30]. Accumulation of NRTI mutations which confers low-level resistance to tenofovir may limit the use of this drug, which is a part of India’s national second line therapy. The results from our study settings, where HIV-1 subtype C is predominant [8] are consistent with those of other studies on subtype C virus from South Africa [28], [29]. In the same setting, we previously also showed that four out of ten patients with NVP and EFV failure accumulated cross-resistance against etravirine [31]. Therefore it is important to identify early virological failure to limit the emergence of cross-resistance to other drugs.

Given limited drug options, patients who have good immunological status with minimal drug resistance and low viremia may still get benefit from their first line regimen as observed by us and others [26]–[29]. In contrast, patients with compromised immunological status and viremia should be switched to second line therapy immediately to limit the emergence of drug resistance and cross-resistance which may jeopardise the second line options. Some of the issues emerging from this finding relate specifically to using multiple approaches to optimise the treatment switch to second line therapy. Patients’ immunological (CD4 count), virological (viral load) and drug resistance data should all be used for optimal treatment switch decisions. Further research on this topic needs to be conducted to determine the role of drug resistance mutations such as M184V, K103N, V106M and Y181C in viral fitness and recognition of cytotoxic T lymphocyte (CTL) and T-helper epitopes within RT, specifically in HIV-1 subtype C strains and their potential role in the therapy response against these HIV-1 subtype C viruses.

A number of caveats need to be noted regarding the present study. First, the number of patients was relatively small. Second, the adherence measurements by self-report may overestimate the outcome. Third, the majority of the patients in this cohort showed good adherence throughout the study period, which may also have influenced the outcome. Therefore our findings may not generalize to other geographical settings in the country, where the adherence is considerably lower. Finally, we are not able to genotype the patients who had viral load below <2000 copies/mL. However a technical merit of our study is that it reflects the natural clinical practices in the setting where the national guidelines are followed.

In conclusion, our study documents for the first time the evidence that among patients who reported perfect adherence to a standardized national first line regimen, the vast majority of the virologically supressed patients continued responding well to the therapy after nearly four years. Our results also strongly support virological monitoring in the setting. It serves not only to detect early virological failure, but can also be used to alert clinicians to the need for targeted adherence interventions for those patients whose viral rebound indicates sub-optimal adherence. The accumulation of new NRTI mutations also supports calls for virological monitoring to identify early failure to avoid the development of cross-resistance to other NRTI drugs. Such cross resistance may jeopardise the national second line options which include two NRTI drugs. Future research should therefore investigate the role of drug resistance and treatment outcome with HIV-1 subtype C viruses to identify the optimal time of switching failing patients to appropriate second line regimens.
